# Computational simulation of vasopressin secretion using a rat model of the water and electrolyte homeostasis

**DOI:** 10.1186/1472-6793-10-17

**Published:** 2010-08-25

**Authors:** Louis Nadeau, Danielle Arbour, Didier Mouginot

**Affiliations:** 1 Centre de recherche du CHUQ (CHUL), Neurosciences and Université Laval, Québec, G1V 4G2, Canada

## Abstract

**Background:**

In mammals, vasopressin (AVP) is released from magnocellular neurons of the hypothalamus when osmotic pressure exceeds a fixed set-point. AVP participates to the hydromineral homeostasis (HH) by controlling water excretion at the level of the kidneys. Our current understanding of the HH and AVP secretion is the result of a vast amount of data collected over the five past decades. This experimental data was collected using a number of systems under different conditions, giving a fragmented view of the components involved in HH.

**Results:**

Here, we present a high-level model of the rat HH based on selected published results to predict short-term (hours) to long-term (days) variation of six major homeostatic parameters: (1) the extracellular sodium concentration, (2) the AVP concentration, (3) the intracellular volume, (4) the extracellular volume, (5) the urine volume and (6) the water intake. The simulation generates quantitative predictions like the daily mean of the extracellular sodium concentration (142.2 mmol/L), the AVP concentration, (1.7 pg/ml), the intracellular volume (45.3 ml/100 g body weight - bw), the extracellular volume (22.6 ml/100 g bw), the urine volume (11.8 ml/100 g bw) and the cumulative water intake (18 ml/100 g bw). The simulation also computes the dynamics of all these parameters with a high temporal resolution of one minute. This high resolution predicts the circadian fluctuation of the AVP secretion (5 ± 2 pg/ml) and defines the limits of a restoration and a maintenance phase in the HH (2.1 pg/ml). Moreover, the simulation can predict the action of pharmacological compounds that disrupt the HH. As an example, we tested the action of a diuretic (furosemide) combined with a sodium deficient diet to generate quantitative prediction on the extracellular sodium concentration (134 mmol/L) and the need-induced water intake (20.3 ml/100 g bw). These simulated data are compatible with experimental data (136 ± 3 mmol/L and 17.5 ± 3.5 ml/100 g bw, respectively).

**Conclusion:**

The quantitative agreement of the predictions with published experimental data indicates that our simplified model of the HH integrates most of the essential systems to predict realistic physiological values and dynamics under a set of normal and perturbed hydromineral conditions.

## Background

Body water is distributed between the intracellular fluid (ICF) and the extracellular fluid (ECF) compartments (interstitial tissue, vascular space), whose volume depends on the osmotic pressure exerted by their electrolyte composition. Due to the selective permeability of biological membranes, sodium (Na+) and accompanying anions are mostly restricted to the ECF compartment, while potassium (K+) is confined to the ICF compartment. Therefore, these two ions are the effective electrolytes creating the osmotic pressure and affecting the movement of water between the two body water compartments. In mammals, the maintenance of osmotic pressure is crucial for the integrity of the cells and organs and small variations from a stable set-point trigger compensatory responses to restore the body fluid osmolality. These homeostatic responses mainly control the retention of water and Na^+ ^at the level of the kidneys [1,2], as well as fluid and Na^+ ^ingestive behaviors [3]. For instance, plasma hyperosmolality triggers rapid homeostatic responses like vasopressin (AVP) secretion, an increased rate of natriuresis and the sensation of thirst [4-7].

The simultaneous quantification of the ECF osmolality and resulting homeostatic responses at a high temporal resolution would be difficult to measure and study experimentally and one approach would be to design a realistic computational model aimed at simulating the dynamics of the biological parameters under study. This model does not have to be necessarily as complex as the biological function it simulates [8]. However, it should include the essential measurable inputs and outputs and connect them in a manner that carries out the processing that occurs, here the secretion of AVP and the adjustment of the ECF osmolality. In this line, the present model of the hydromineral homeostasis is organized around three distinct systems that define the scope of the simulation: (1) The current state of the ICF and ECF compartment in terms of ion composition and volume; (2) The input/output to the body fluid compartments in terms of regulated and unregulated water and Na^+ ^intake or loss, respectively and (3) The controllers that specifically regulate water and Na^+ ^excretion at the level of the kidneys. Note that some of the biological parameters that characterize each of the systems rely on necessary assumptions that are presented in the methods (see the model architecture).

The modeling principle is based on the use of "high-level" functions, i.e., a black-box approach to model the components of each system. Such a modeling approach uses necessary simplifications of the model and does not therefore implement all the complex cascade of hormones and/or cellular mechanisms underlying the output of the black boxes. For instance, the model includes the "aldosterone (ALD) controller" and the "atrial natriuretic peptides (ANPs) controller", which add to the model the capacity to respond to a certain degree of hypovolemia and hypervolemia, respectively by modulating Na^+ ^excretion. The secretion of ALD and ANPs is multifactorial and depends on the action of other hormones like angiotensin II and oxytocin [9]. However, considering the experimental data on ALD and ANPs will indirectly include the regulatory action of angiotensin II and oxytocin in this high-level model without a direct implementation of the regulatory action of these hormones in the model. Moreover, the experimental data on ALD, ANPs and AVP introduce in the model the relationship that links the secretion of these hormones to the ECF volume without the need of implementing a complex system of blood pressure control.

The purpose of the present study was to develop a computational simulation of the dynamics of the ECF osmolality and vasopressin release under stable hydromineral condition, as well as during osmotic challenges.

## Methods

### Biological parameters

The present model will specifically be developed for rats since this animal species has historically provided much of the data in the field of the hydromineral homeostasis.

All of the initial values for the biological parameters used in the simulation were compiled from published experimental data and are presented in Table 1. Biological parameters are tightly correlated with the animal's weight. However, a simple linear scaling of the parameters extracted from the literature may be biased by physiopathological condition of the animals under study. For example, obese rats have an expanded skin surface compared to lean rats, a condition that directly affects the amount of water lost by evaporation. In the same line, the amount of metabolic water resulting from the ingested food in obese rats is larger than in lean rats [10]. Therefore, to minimize the introduction of conversion error in the model, the rat's weight was fixed at 255 g, a value that is well within the range of weights reported in the experimental studies referenced. Moreover, data were selected from studies using rats in the same age range because aging directly affects the water balance of rats [11].

**Table 1:** List of the parameters and their physiological value used in the simulation.

Parameters	Symbols	Initial values	Physiological values and references
ICF potassium concentration	[K^+^_ICF_]	112 mmol/L	112 [12]

AVP concentration	[AVP] (t = 0)	1 pg/ml*	≈1 [19]; 1.05 ± 0.4 [11]; 1.7 ± 0.3 [23]; 1.9 ± 1.1 [46]

ECF Na^+ ^concentration	[Na^+^_ECF_] (t = 0)	140 mmol/L*	≈135 [17]; 138 ± 2.6 [22]; 143.3 ± 0.4 [37]; 145.6 ± 1.2 [23]); 146 ± .8 [47]

ECF volume	ECF_V _(t = 0)	23 ml	23 [12]

ICF volume	ICF_V _(t = 0)	46 ml	46 [12]

Rat weight	W	255 g*	150-250 [20]; 210-230 [23]; 245 ± 5 [24]; 250-280 [47]; 250-325 [48]; 262 ± 15 [22]

Sodium clearance	C_Na+_	0.0086 ml/min	See Additional file 2[21-24]

The values identified with a star (*) were defined by fine-tuning the simulation within the range of the data reported in the literature. (t = 0) indicates the initial set of the parameter to start the simulation. The value of all these time-dependent parameters is updated every minute during the simulation.

### Software

The simulation is performed using Scicos, a dynamical system simulator included in the Scilab software http://www.scilab.org. Scilab is an open source software that is equivalent to Matlab, the leading software in scientific computing (MathWorks - http://www.mathworks.com). Scicos has a graphical user interface (GUI) that is particularly useful for our task of modeling a complex system and the flexibility of the software allows easy substitution of a standard box by a function coded in a different programming language (Fortran or C). The simulation has a time resolution of one minute implying that time-dependent parameters are updated every minute according to the current state.

### The model architecture

**Figure 1:**
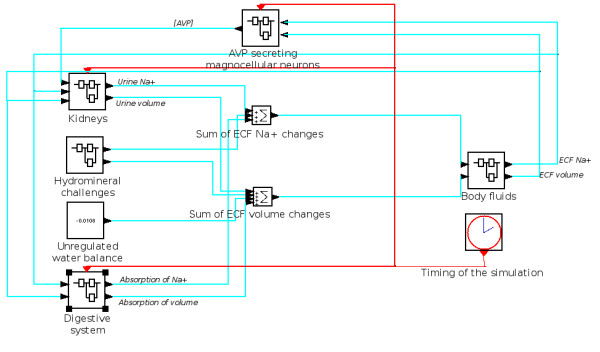
**Screenshot of the Scicos' simulation software displaying the architecture of the simulation**. Each meta-box represents a component of the system. Blue arrows indicate the direction flow of the physiological parameters during the simulation. Red arrows carry the time signal of the simulation (1 min).

The "high-level" simulation is organized around three functional systems: (1) the current state of the body fluid compartments, (2) the controllers specifically acting at the level of the kidneys and (3) the inputs/outputs of the body fluid compartments. For the implementation of the model in the computer simulation, each system is represented by one or several black boxes, called meta-boxes. The system "current state of the body fluid compartments" is composed of one meta-box called "body fluids". The system "inputs/outputs of the body fluid compartments" is composed of four meta-boxes called "kidney", "digestive system", "unregulated water balance" and "hydromineral challenges". Finally, the system "controllers" is composed of one meta-box called "AVP secreting magnocellular neurons" and additional corrections for active natriuresis and/or Na^+ ^retention. In addition, the simulation includes a timing generator (red clock) and two boxes that summate the sodium changes and the water changes, respectively. Figure 1 illustrates a screenshot of Scicos' main window, displaying the architecture of the simulation described above. The content of all the meta-boxes are detailed in the following sections

#### System 1; the meta-box "body fluids"

The body fluids are distributed between the ICF and the ECF compartments. The solute composition of each compartment differs considerably: Na^+ ^is the major cation in the ECF, whereas K^+ ^is dominant in the ICF. In the present simulation we simplified the ECF compartment by merging together the vascular space and the interstitial fluid because the time course describing Na^+ ^exchange between these two distinct spaces is below one minute [12], the time resolution of the simulation. The ICF K^+ ^concentration ([K^+^]) is set as a non-variable parameter in the simulation [12]. This assumption was initially chosen because the large volume of the ICF compartment prevents dramatic changes in the [K^+^]. In addition, regulation of [K^+^] was considered less critical for the integrity of the organism than regulation of [Na^+^] [6]. Therefore, time-dependent changes in ICF volume in the simulation result from water movement driven by changes in ECF [Na^+^].

The ECF [Na^+^] was preferred over the ECF osmotic pressure because osmotic pressure depends on additional parameters including the level of sugars, proteins and urea. However, a correction was included in the simulation to account for the osmotic weight of these parameters (see next section).

Altogether, the body-fluid compartments are simulated by a meta-box that dynamically integrates three biological parameters: (1) the ECF volume, (2) the ECF [Na^+^] and (3) the ICF volume. The physiological set-point of these parameters is indicated in Table 1.

#### System 2; the meta-box "AVP secreting magnocellular neurons"

This meta-box is the main controller of the simulation. It simulates the regulation of AVP concentration in the plasma and it contains three items: (1) a mathematical equation adjusting the AVP concentration as a function of the ECF osmolality, (2) a time-dependent degradation of AVP and (3) a circadian modulation of AVP secretion.

(1) The mathematical equation was created using experimental measurement from Dunn et al., 1973. In this paper and other reports, it is shown that the AVP level is a linear function of the plasma osmolality and an exponential function of the volume [13-16]. In addition, a correction was included in the equation to reflect the volume-dependent change in the slope of AVP secretion reported in the experimental data. Using a linear regression based on Figure six of Dunn et al. (1973), the correction term of the slope was set at 11. The ECF [Na^+^] in the equation was also corrected for the contribution of sugar, proteins and urea to plasma osmolality according to the approximation stated in Verbalis, 2003, but using the values for the rat (14 mosmol/L) [17]. The resulting AVP level is calculated using the following equation:

(1) $$\begin{array}{l} \left[AVP(t)\right]=(0.91-\frac{11\cdot ECF_{\Delta V}(t)}{ECF_{V}(t=0)})\cdot (200\cdot ([Na_{ECF}^{+}(t)]\\ -0.1375))+1.3\cdot e^{\frac{-17\cdot ECF_{\Delta V}(t)}{ECF_{V}(t=0)}} \end{array}$$

All symbols are defined in Table 1. The Delta (Δ) symbol denotes the difference between the current state in the simulation and the initial state defined in Table 1. All the numerical values are based on the fits of the experimental data reported in Dunn et al. (1973). Since a negative value for hormone secretion is meaningless, we avoid non-physiological values by allowing "ECF_ΔV_(t)" to be strictly negative (or zero) in the equation and by forcing AVP level to be positive (or zero). Note that the change in AVP secretion caused by osmotic fluctuation is larger than the change caused by volume fluctuation (see Additional file 1).

(2) A time-dependent degradation of AVP was added to the model so that 15% of circulating AVP is cleared every minute when AVP secretion is reduced [18] (see discussion).

(3) A circadian pattern of AVP secretion is also included in the model. This implementation was derived from data presented in Figure one of Graugaard-Jensen *et al*. (2006). Thus, the total AVP concentration is linearly reduced at a speed of 5%/h starting at 2 AM to reach a maximum of 30% reduction at 8 AM. Then, the negative modulation of AVP secretion is progressively inactivated from 8 AM to 11 AM before being controlled by the current state of the ECF [Na^+^] (see discussion).

#### System 3; the meta-box "Kidney"

This meta-box is designed to dynamically simulate the rate of urine flow and the rate of Na^+ ^excretion by the kidneys (passive Na^+ ^clearance and active natriuresis or Na^+ ^retention).

The rate of urine flow is calculated every minute in the simulation. The calculation is adapted from an equation that expresses the rate of urine flow as a function of AVP in humans [6]. Here, the basal AVP level was linearly rescaled from 1 pg/ml (mean basal level in Humans [19]) to 2.3 pg/ml (mean basal level in rats [20]). The rate of urine flow was also scaled so that the flow caused by 2.3 pg/ml of AVP corresponds to 6 μl/min/100 g of body weight (bw). This value corresponds to the normal rate of urine flow in the rat [17]. The maximum rate of urine flow, in the absence of AVP, was fixed at 138 μl/min/100 g bw that corresponds to 80 ml/day/100 g bw [19].

The value of Na^+ ^clearance used in the model (table 1) was calculated from experimental data reported in four distinct studies [21-24] and calculations are presented in Additional file 2. Na^+ ^clearance combined with the action of the AVP controller are however, not sufficient to overcome the small variations in ECF volume resulting from the change in ECF osmolality (data not shown). We thus, implement two additional controllers in the model to introduce a volume-dependent change in Na^+ ^excretion (active natriuresis) or in Na^+ ^retention. These active controllers will be referred to as the atrial natriuretic peptides (ANP) factor and as the aldosterone (ALD) factor, respectively. ANP_factor _is a multiplicative factor (≥1) on Na^+ ^excretion that accounts for an increase in ECF volume over the volume set-point (table 1). ALD_factor _is a multiplicative factor (≤1) on Na^+ ^excretion that accounts for a decrease in ECF volume below the volume set-point.

The rate of Na^+ ^excretion is calculated every minute with the following equation:

(2) $$Na_{urine}^{+}=\left[{\text{Na}}_{\text{ECF}}^{+}\right]\cdot C_{Na^{+}}\cdot ALD_{factor}\cdot ANP_{factor}$$

Na^+^_urine _refers to the millimoles of Na^+ ^excreted during the current minute of the simulation. [Na^+^_ECF_] is the ECF [Na^+^] one minute before the current minute. C_Na_^+ ^is the Na^+ ^clearance defined in Table 1.

ANP_factor _was calculated from Paul et al. (1988) in two separated steps: (1) The plasma ANP concentration was first computed as a function of the expansion of the plasma volume (from Figure four in Paul *et al*., 1988) and a linear interpolation was added between the experimental values. The maximum ANP level was set to 2000 pg/ml (the upper limit appearing in Figure four of Paul *et al*., 1988) to prevent a non-linear error in case of large volume changes. (2) The increase (in percent) in natriuresis was associated with the plasma ANP concentration (from Figure five in Paul *et al*., 1988) and this percentage is used as the ANP_factor_.

ALD_factor _was based from two studies [25,26]. Figure oneB of Stricker et al. (1979) was first adapted to express the aldosterone concentration as a function of the ECF volume instead of the plasma volume. The study of Morris et al. (1973) was then adapted to compute ALD_factor _as a function of the aldosterone concentration. Here the dose of aldosterone (μg) was converted to aldosterone concentration by dividing the dose by the volume of plasma. Moreover, a linear interpolation including an ALD_factor _with an upper limit of 1 (for aldosterone concentration of 0 ng/100 ml) and a lower limit of 0.2 (for aldosterone concentration of 200 ng/100 ml) was introduced to the conversion of the experimental data.

#### System 3; the meta-box "Digestive system"

This meta-box is created to simulate water and Na^+ ^intake. It contains a digestive module and a motivation module making this meta-box the most complex of the model. The "digestive module" simulates the transition of orally ingested water from the stomach to the intestine and its final absorption in the ECF. This module reproduces the intestine and stomach modules generated by Toates *et al*. (1970), which was based on experimental data from two studies [27,28]. Our model includes the same time constants for (1) the active and passive exchange of water and Na^+ ^between the stomach, intestine and ECF, and (2) the emptying of water content from the stomach to the intestine.

The motivation module takes the decision of drinking distilled water or Na^+^-containing water. These decisions are based on two simple rules that are sufficient to reproduce realistic behavior.

*Rule 1*: If ECF [Na^+^] exceeds the pre-established set-point by a threshold of 4% [5,29], the motivation module allows intake of distilled water until the ECF [Na^+^] is restored, or until the stomach is full (volume of 5 ml). In the latter case, water intake stops until the stomach volume is reduced to a comfortable level (3.1 ml) by emptying water into the intestines and by passive diffusion to the ECF.

*Rule 2*: If ECF [Na^+^] is normal (142 mmol/L) or slightly lower, the motivation module allows intake of Na^+^-containing water (50 mmol/L or 0.28% of Na^+^) to model unregulated drinking [30]. In the simulation, unregulated drinking is triggered randomly following a statistical distribution, which is a translated sinusoidal to account for the circadian modulation of fluid and electrolyte intake.

There is no access to dry food in the model and the introduction of Na^+^-containing water compensates for the lack of this essential source of Na^+^. The amount of Na^+ ^contained in the water is adjusted to approximately match the daily amount of Na^+ ^ingested by a rat of 255 g fed with regular pellets (0.3% Na^+^). Instead of randomly injecting Na^+ ^in the model, the sodium input is associated with water input since in reality the feeding and drinking period are associated in rat.

#### System 3; the box "Unregulated water balance"

This box includes a constant factor that accounts for the balance between the loss of water in sweat (22 ml/day) and feces (4 ml/day) versus water gained from eating food (2.6 ml/day) and metabolic processes (7.9 ml/day). Reference values were obtained from [10] and were linearly scaled for a rat of 255 g. In the simulation, the water balance is negative (-0.0108 ml/min or -15.5 ml/day) implying that the gain of water from food and metabolic process is not sufficient to offset the loss of water from sweat and feces. Therefore, the water intake through drinking has to overpass the urine volume to prevent the loss of body fluid.

#### System 3; the meta-box "Hydromineral challenges"

This box contains two distinct hydromineral challenges that can be switched on during the simulation. All of the virtual challenges occur during the sleeping period of the rat, a period during which most of the experimental protocols are performed in laboratories. Simulation of hydromineral challenges, inducing either intracellular or extracellular dehydration, are intended to predict changes in the ECF [Na^+^] and volume, in the ICF volume as well as the changes in AVP secretion.

##### Challenge 1: intracellular dehydration

This virtual challenge simulates the effect of a short-term (10 minutes) jugular injection of 1 mmole Na^+^. This injection of Na^+ ^*per se *(without water) was intended to mimic a rise in extracellular osmolality creating an intracellular dehydration. This challenge evokes AVP secretion and osmotic thirst leading to water intake.

##### Challenge 2: extracellular dehydration

This challenge simulates the effect of a loop diuretic (furosemide) that is known to induce a rapid urinary loss of Na^+ ^and water. The resulting loss of fluid stimulates AVP secretion and hypovolemic thirst leading to water and salt intake. The effects of furosemide were simulated by substituting the regular kidney output of the model (see meta-box "kidney" above) by a computed furosemide output. This specific output consisting of a modified urine flow rate and [Na^+^] induced by the furosemide injections were calculated from two studies reporting the dynamics of these parameters after furosemide treatment [31,32]. Linear interpolation of the data illustrated in Figure one in Kikkoji *et al*. (1988) and in Figure one in Hori *et al*. (1988) served to calculate the urine flow rate and [Na^+^], respectively.

### Experimental measurements

Experiments were carried out according to the recommendations of the Canadian Council on animal care and approved by the Ethical Committee on Animal Research of the Université Laval.

**Figure 2:**
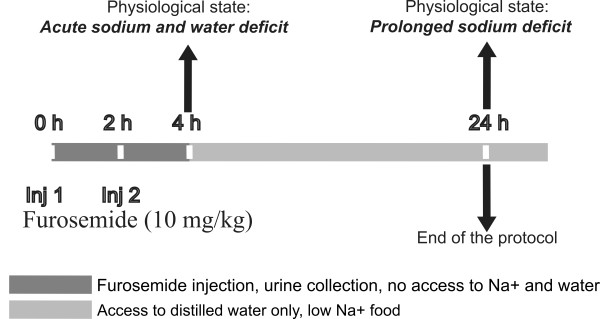
**Schematic representation of the experimental protocol used to induce extracellular dehydration**. 4 h (dark gray bar) indicates an acute water and Na^+ ^deficit created by two injections of furosemide (0 h, inj. 1 and 2 h, inj. 2, respectively). 24 h (light gray bar) indicates a sustained Na^+ ^deficit and rehydration created by *ad libitum *access to tap water and Na^+ ^deficient food. Urine volume and water intake were measured at 4 and 24 h.

Experimental data were collected from 18 male Wistar rats (8 weeks postnatal) to validate the predictive results obtained with the simulation of extracellular dehydration (furosemide injections). The experimental protocol combines two subcutaneous injections of furosemide (10 mg/kg) and a Na^+ ^deficient diet (0.01-0.02% NaCl; TD 90228, Harlan Teklad, WI) that extends over 20 h. Figure 2 illustrates the timeline of the experimental protocol where time 0 in the experimental protocol corresponds to 8 AM in the simulation. During the first 4 h, the rats were housed individually in metabolic cages without access to water and food. At time 0 (8 AM), the rats received the first furosemide injection, followed by the second injection 2 h later (10 AM). At time 4 h (12 AM), urine was collected and the urine volume was measured. The rats were housed individually in regular plastic cages where they had access to tap water (0.001% sodium) and to Na^+ ^deficient food for 20 h. In the simulation the rat did not have access to food and the lack of this Na^+ ^source was compensated by water containing Na^+^. The Na^+ ^content was adjusted to match the amount of Na^+ ^ingested during exposure to the Na^+ ^deficient food (≈2 mg/100 g bw).

## Results

The following section presents the computed predictions of the dynamics of six parameters of interest: the body fluid compartments (ECF [Na^+^] and volume; ICF volume), the AVP level and the water intake and excretion under balanced hydromineral conditions (control) and during two evoked hydromineral challenges.

### Computed predictions under control hydromineral conditions

Here, the control conditions refer to a rat being active during the night and sleeping during the day. The rat has free access to water and sodium by bouts, randomly and mostly distributed during the night. These control conditions in our simulation are not "steady-state" simulation. Indeed constant inputs and outputs do not represent physiological conditions for a rat (except under anesthesia and continuous water and salt perfusion). The simulation under control conditions quantifies the mean level of the six parameters of interest for 24 h, over a period of 20 consecutive days. The mean and standard deviation of each parameter over that long-term simulation are presented in table 2. Note that the predicted ECF [Na^+^], ECF and ICF volume remain stable over the long-term simulation and agree with the physiological expectations. Beyond these global data, the simulation predicts significant differences between the sleeping period (8 AM to 8 PM) and the waking period (8 PM to 8 AM) of the rat over the period of 20 consecutive days. The low probability of unregulated drinking that occurs during the sleeping period is correlated with a weak cumulative water intake (12 ± 4 ml) and a moderate increase (2.1%) in ECF [Na^+^]. Indeed, the predicted maximal ECF [Na^+^] for this period is 145 ± 1 mmol/L compared to the daily mean ECF [Na^+^] that includes both the sleeping and the waking period (142 ± 1 mmol/L; paired t-test, p < 0.001, n = 20 days). The predicted ECF volume (daily mean: 58 ± 2 ml) reaches a minimum of 54 ± 2 ml (paired t-test, p < 0.001, n = 20 days) and the ICF volume (daily mean: 116 ± 1 ml) shows a minimum of 113 ± 1 ml during the sleeping period. These changes are correlated with a progressive increase in AVP, which reaches a maximal level of 7 ± 4 pg/ml compared to the daily mean AVP level of 3 ± 2 pg/ml (paired t-test, p < 0.001, n = 20 days). During the sleeping period, the urine flow is reduced from 0.021 ± 0.006 ml/min (daily mean) to 0.014 ± 0.004 ml/min (paired t-test, p < 0.001, n = 20 days) and the urine [Na^+^] is increased from 80 ± 25 mmol/L (daily mean) to 91 ± 27 mmol/L (paired t-test, p < 0.001, n = 20 days)

**Table 2:** Long-term predictions of the physiological parameters of the simulation.

Parameters	Mean	SD	Physiological values and references
Mean ECF Volume (ml/100 g)	22.6	0.6	≈21.9** [10]; 22.3 ± 0.8 [49] ≈23.7** [50]; ≈23.1 [12]; 23.2 ± 2.4 [51]; 24 ± 2 [52]

Mean ECF [Na+] (mmol/L)	142.2	0.7	138 ± 2.6 [22]; 140 ± 3 [53]; 141 ± 2 [46]; 141.6 ± 0.3 [54]; 142 ± 1 [55]; 143.3 ± 0.4 [37]; 129 to 150 [17]

Mean AVP (pg/ml) during the maintenance phase	1.7	0.9	1.1 ± 0.21 [56]; 1.7 ± 0.3 [23]; 1.8 ± 0.3 [54]; 1.9 ± 1.1 [46]; 2.3 ± 0.9 [20]; 2.5 ± 1.3 [53]

Urine Volume (ml/100 g)	11.8	3.4	10.8 ± 3.6 [57]; 8.9 ± 5.9 [17]; 13 [19]; 13 ± 2 [58]; 14 ± 1 [34]

Water intake (ml/100 g)	18.0	3.7	15.2 ± 1.8 [10]; ≈16 [38]; 20 ± 3 [58]

Na+ intake (mmol/100 g)	0.69	0.17	≈0.75 [59]; 0.84 ± 0.02 [34]; 0.97 ± 0.14 [23]

Mean ICF Volume (ml/100 g)	45.3	0.3	≈ 45.9 [12]; 45 ± 1 [50]**; 44.5 ± 3.1** [51]; ≈ 44.6 [10]; 50 ± 5** [52]

Daily mean and standard deviation of each parameter are obtained after 20 consecutive days of simulation under control conditions. All the predictions are coherent with experimental data reported in the literature according to their deviations. Some experimental data had to be normalized for comparison. The "**" symbol indicates that the value was obtained by subtracting the ICF or ECF volume of the model from the total water content of the rat measured experimentally.

**Figure 3:**
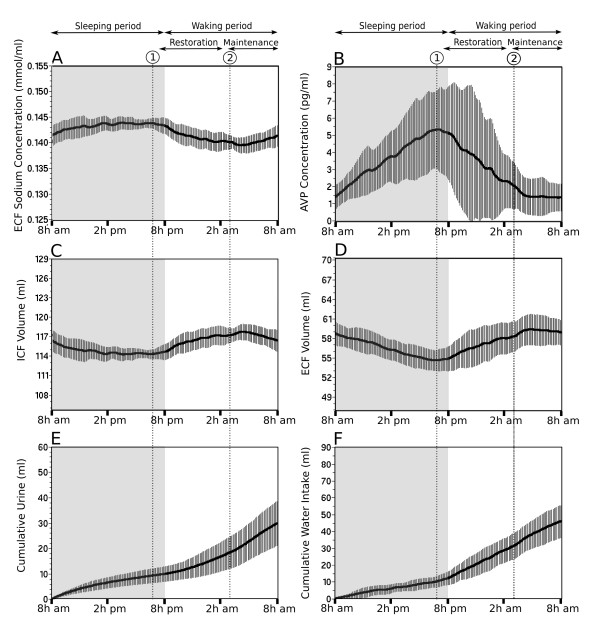
**Circadian predictions of the vasopressin release and related physiological parameters generated by a simulation of 20 consecutive days**. In the current rat model, the hydromineral status of the body fluid compartments is characterized by the ECF [Na^+^] (A), the ICF volume (C) and the ECF volume (D). The Inputs/outputs are the cumulative water intake (F) and cumulative urine (E), respectively and the systemic AVP concentration is the controller of the homeostasis (B). In each panel, the value of the parameter is calculated every minute and the solid black line is the mean of the parameter. The vertical bars are the standard deviation. The gray area illustrates the sleeping period of the rat (day) and the white area, the waking period (night). The prediction of the dynamic of the parameters highlights three distinct phases in the circadian fluctuation of the hydromineral balance: (1) the sleeping phase characterized by a progressive rise in ECF [Na^+^] and AVP, (2) the restoration phase (onset at the dotted line 1) covers the recovery of AVP from its peak to basal level and (3) the maintenance phase (onset at the dotted line 2) is characterized by the low variability of AVP level and a restored ECF [Na^+^] and volume.

Interestingly, the high resolution of the long-term simulation (one minute) allows an instantaneous average of each parameter value over the period of the simulation (20 days). This average reduces the inter-day fluctuation of the parameters and highlights the overall trend of the circadian dynamic of the parameters (Figure 3). It clearly indicates that waking period is split into two distinct phases defined here as the restoration phase and the maintenance phase. The onset of the restoration phase corresponds to the peak of AVP level (5 ± 2 pg/ml) and to the maxima of ECF [Na^+^] (144 ± 1 mmol/L; Figure 3A and 3B, dotted line 1). The simulation predicts that restoration of AVP concentration (Figure 3B) requires cumulative water absorption of approximately 22 ml (Figure 3F) and has a duration of approximately 8 h. The maintenance phase follows the restoration phase. The onset of this phase corresponds to the time point at which the current AVP concentration is not significantly different from its steady-state concentration observed between 2 and 8 AM. Using the confidence interval of this period (1.2 pg/ml to 2.1 pg/ml, with a confidence level of 95%), the onset of the maintenance phase is set to 2.1 pg/ml of AVP (Figure 3B, dotted line 2). At the beginning of this phase the ECF [Na^+^] is restored to a value of 140 ± 1 mmol/L (Figure 3A, dotted line 2). The maintenance phase is essentially composed of unregulated drinking that reflects the mean Na^+ ^and water consumption expected for a rat of about 255 g. The maintenance phase is characterized by a weak variability of the AVP level and a relative stability of the parameters of the body fluid compartment.

### Validation of the model by experimental data

It is essential to validate the predictions of the simulation produced under balanced hydromineral condition. This step is crucial to insure that the prediction of each parameter of interest over a period of 24 h agree with physiological expectations and are supported by published experimental data. However, these experimental data have to fulfill criteria for compatibility with the present model: They have to be sampled from adult animals and from rat strain that do not present physiopathological conditions (Wistar, Sprague-Dawley and Long-Evans rat strain). The animals have to be housed in regular laboratory conditions (*ad libitum *access to regular food diet and water, 12 h circadian cycle, normal temperature). Table 2 summarizes the prediction of the mean and standard deviation of each parameter of the simulation tested over a period of 20 consecutive days and it presents experimental data collected from many studies, which are in agreement with the simulated parameters.

In addition to the validation of the daily means over a long-term simulation, it is important to validate the high-resolution (one minute) dynamic of the simulation. The lack of experimental data approaching the present temporal resolution is critical. However, few studies did collect data with a high enough resolution to validate the simulated dynamics [33-36]. Additional file 3 compares the dynamics of the water intake (input), urine volume (output) and AVP concentration (controller) with these experimental data. The goodness of fit of our simulated parameters was considered satisfactory if the mean of the experimental data is included within the standard deviation of the simulated parameter for 24 h. Additional file 3 indicates that the simulated dynamic of water intake, urine volume and AVP concentration are in agreement with published experimental data measuring these three parameters at several time points during 24 h.

### Role of early water intake in reducing variability of ECF [Na^+^] and circulating AVP level

**Figure 4:**
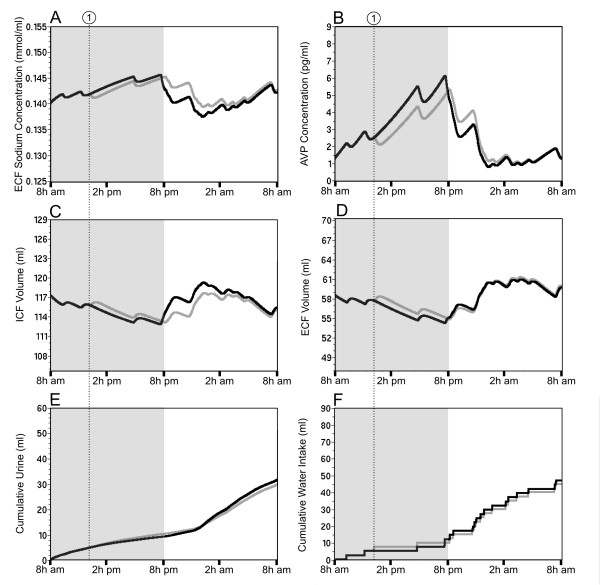
**Predictions of the AVP secretion depends on the fluid intake probability during the sleeping period**. 24 h simulation of the hydromineral parameters is displayed under two distinct conditions. The solid black line illustrates the prediction of the AVP secretion and related hydromineral parameters when fluid intake occurs twice at the beginning of the sleeping period (F, control condition). The solid gray line illustrates the prediction of the hydromineral parameters when the rat was forced to take one additional bout of water (F, dotted line 1).

Data summarized in table 2 highlight the presence of large daily fluctuation in fluid and electrolyte intake over the long-term (here 20 days). These differences mainly depend on the frequency and distribution of unregulated drinking in the simulation (see the meta-box "digestive system in methods) and illustrate the random nature of the daily fluid and electrolyte consumption of the rat. Here the simulation tested the influence of this random consumption on the level and dynamic of the ECF [Na^+^] and AVP for a period of 24 h. The occurrence of a single additional drinking bout (forced drinking) at the onset of the sleeping period (Figure 4F, dotted line 1) delays the increase in ECF [Na^+^] (Figure 4A, gray line), compared to the absence of additional drinking bout (Figure 4A, black line). In this example, the additional drinking bout reduces the circulating level of AVP by about 1 pg/ml (Figure 4B) and shortly delays the onset of the maintenance phase (Figure 4B). The fluctuation of both the ECF and ICF volume are also attenuated (Figure 4C and 4D). These predictions highlight the high sensibility of the system to random daily fluctuation in the frequency and distribution of drinking bout.

### Role of the ICF compartment in reducing variability of ECF [Na^+^] and circulating AVP level

**Figure 5:**
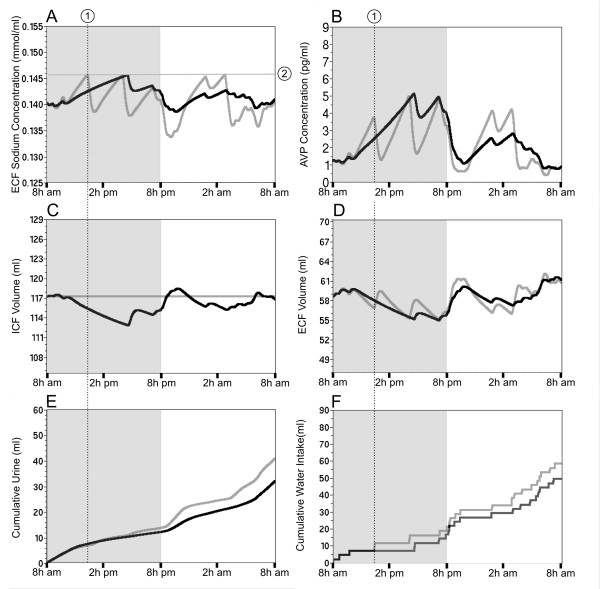
**The ICF compartment is essential for the stabilization of the ECF parameters and AVP secretion**. The solid black line illustrates the prediction of the hydromineral parameters over a 24 h simulation when water exchange is permitted between the ECF and the ICF compartments (C, control condition). The solid gray line illustrates the same prediction when water exchange between the ECF and the ICF compartments is not permitted (C). In such a simulation, the ECF [Na^+^] quickly overpasses the threshold for drinking (A, dotted line 2). The additional need-induced water intake (four threshold crossing, A, dotted line 2) increases the total water intake by approximately 19% (F). Note that the absence of water exchange between the two compartments is characterized by larger fluctuations in the ECF [Na^+^] (A) and volume (D), as well as in AVP secretion (B).

The long-term simulation predicts a circadian fluctuation in ICF volume, likely indicating a putative role of the ICF compartment in stabilizing the ECF [Na^+^]. In order to validate that hypothesis, we simulated a lack of water transfer between the ICF and ECF fluid compartments for a period of 24 h (Figure 5C, solid gray line). Interestingly, this test predicts a sharp increase in ECF [Na^+^] during the sleeping period that rapidly exceeds the threshold for drinking (4%, Figure 5A, line 2) and triggers water intake (Figure 5F, dotted line 1). Moreover, the occurrence of sharp rise in ECF [Na^+^] over the threshold is also increased, triggering additional need-induced water intake to restore ECF [Na^+^] (Figure 5F). The simulation also predicts increased AVP level fluctuation (Figure 5B) that contributes to attenuate fluctuation in ECF [Na^+^]. It also appears from this prediction that drinking bouts occurring at the end of the sleeping period and during the waking period (Figure 5F) trigger fluctuation of larger amplitude in the ECF [Na^+^] (Figure 5A) and circulating AVP level (Figure 5B). The water intake needed to compensate for the lack of ICF water transfer is approximately 9 ml over a period of 24 h, representing 19% of the daily water intake. The fluctuations in ECF [Na^+^], circulating AVP level and drinking bouts are greatly attenuated when the ICF compartment is included in the simulation (Figure 5, solid black line), emphasizing the buffer role of the ICF compartment, working in synergy with circulating AVP to maintain the stability of the ECF [Na^+^] and to lower the daily need of water intake.

The previous results demonstrate that this model of rat hydromineral homeostasis produces coherent predictions of the major parameters of the system under balanced hydromineral conditions indicating that all essential components are included in the model. The next series of simulations are intended to predict changes in these parameters in response to hydromineral challenges.

### Challenge 1: Intracellular (osmotic) dehydration

**Figure 6:**
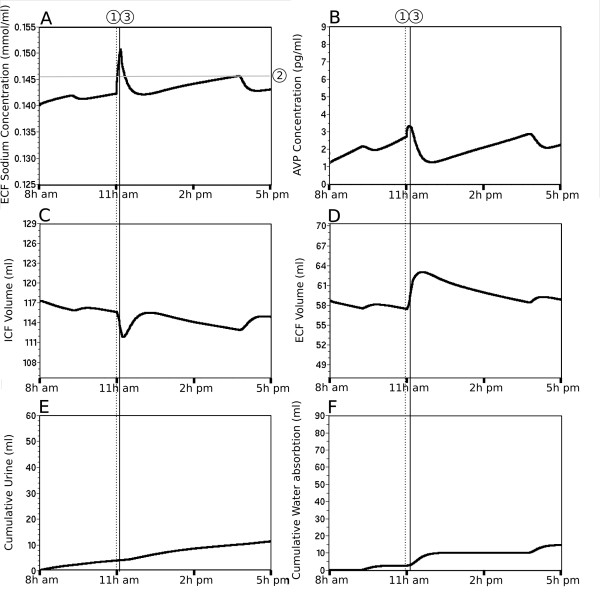
**Simulation of intracellular dehydration**. The dehydration is induced by a 10 min injection of hypertonic saline (1 mmol Na^+^). Dotted line 1 indicates the onset of the rapid homeostatic responses, i.e. the reduction in the intracellular volume (C) and the increased AVP release (B). The need-induced water intake (F) is further engaged when the ECF [Na^+^] overpasses the threshold for drinking (A, dotted line 2). Solid line 3 indicates the first drinking bout induced by the dehydration. Note that the decrease in AVP level (B) is correlated with the increased water absorption at the level of the digestive tract (F).

The challenge consists of injecting 1 mmol Na^+ ^over 10 minutes into the ECF compartment. The Na^+ ^infusion abruptly raises the ECF [Na^+^], creating a threatening hyperosmotic condition (Figure 6A). The simulation indicates that the responses engaged to restore the ECF [Na^+^] level occur in two distinct phases contributing to an increase in the ECF volume (Figure 6D). The first phase consists of a rapid transfer of water from the ICF to the ECF compartment (Figure 6C, dotted line 1). The reduction in the ICF compartment reaches 3% of its initial volume in a short interval, causing intracellular dehydration. Na^+ ^infusion also triggers an immediate rise in AVP levels that is added to the initial increase resulting from the reduced probability of drinking during the sleeping period (Figure 6B). The peak in AVP (3.2 pg/ml) reduces the urine flow rate to optimize water conservation and lower the ECF [Na^+^]. Note that the rise in AVP is concomitant to 5.5 fold increased in urine Na^+ ^concentration (data not shown). The second corrective phase consists of water absorption (Figure 6F, line 3). This phase is shortly delayed and is engaged after the ECF [Na^+^] exceeds the pre-established threshold (Figure 6A, line 2) and after the stomach starts exchanging water with the ECF and the intestine. The intake of distilled water also contributes to the increase in ECF volume and also acts to restore the ICF volume. The rapid dilution of the extracellular Na^+ ^by exchanging water with the ICF, by ingesting water and by increasing renal Na^+ ^excretion are responsible for the quick recovery of the ECF [Na^+^].

### Challenge 2: Extracellular (hypovolemic) dehydration

**Figure 7:**
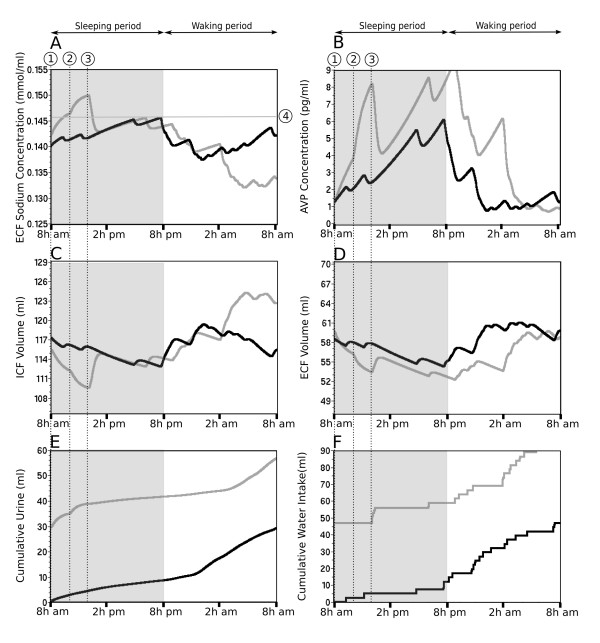
**Simulation of extracellular dehydration**. Extracellular dehydration is preceded by a 24 h simulation performed under normal hydromineral conditions (control condition; A to F, solid black line; day 0). The simulated dehydration protocol is induced by two injections of furosemide along with a Na^+ ^deficient diet (A to F, solid gray line, day 1). Cumulative urine excretion (E) and cumulative water intake (F) predicted at the end of day 0 are reported at the start of day 1. During the hydromineral challenge, the first injection of furosemide is given at the beginning of the simulation (8 AM, dotted line 1) and the second injection is given at 10 AM (dotted line 2). *Ad libitum *access to tap water is only permitted after the first 4 h of the simulation (dotted line 3) and the solid line 4 (panel B) indicates the threshold for drinking.

The challenge consists of two injections of furosemide without access to water during the first 4 h. That depletion period is followed by 20 h of a Na^+ ^deficient diet and *ad libitum *access to tap water. The challenge starts at 8 AM (day 1) after one day of simulation under balanced hydromineral conditions (day 0). The 24 h pre-simulation is intended to generate hydromineral parameters that fluctuate slightly from their pre-established set-point. The dynamics of these parameters are presented in Figure 7 as a control (solid black line). Note that cumulative urine volume and water intake at the onset of day 1 are thus, above 0 (Figures 7E, F, respectively). As expected, furosemide injections rapidly increase the cumulative urine excretion by 10.5 ml (35% of the cumulative urine volume; Figure 7E, gray line) decreasing the ECF volume by approximately 10% (Figure 7D). AVP shows a sharp and massive release (Figure 7B, gray line) with a peak of 8.2 pg/ml during the furosemide protocol. AVP release reduces the urine flow rate to a minimum (0.5 μl/min predicted after the first 4 h of the simulation). Interestingly, the simulation also predicts an increased ECF [Na^+^] of approximately 6% (Figure 7A) due to the negative balance between water and Na^+ ^loss. Note that the rise in [Na^+^] exceeds the threshold (Figure 7A, line 4) and would have triggered drinking if water was available. The ICF volume is simultaneously reduced by 5% (Figure 7C) to buffer the increase in ECF [Na^+^]. Predictions of all the physiological parameters after 4 h of extracellular dehydration are summarized in Table 3 and compared to the control day. The furosemide treatment induces mild hypovolemia and the simulation predicts water intake as soon as water is available (Figure 7F). Note the large water intake despite a low unregulated drinking probability during the sleeping period. The simulation predicts that water intake combined with the Na^+ ^deficient diet set after the injections progressively reduces the ECF [Na^+^] to 134 mmol/L (Figure 7A), resulting in hyponatremia. The progressive decrease in ECF [Na^+^] is correlated with low level of ANP and a high level of ALD to minimize renal Na^+ ^excretion (0.91 μmol/min after the first 4 h vs. 1.08 μmol/min at the end of the simulation). Moreover, water intake restores both the ICF and ECF volume. However, if repletion of the ECF volume is completed by the end of day 1 (Figure 7D, gray line), the simulation predicts an expansion of the ICF volume (Figure 7C, gray line). The overload of the ICF compartment likely results from the predicted low ECF [Na^+^] causing an osmotic movement of water to the ICF compartment. Predictions of all of the physiological parameters after 24 h of sodium depletion are summarized in Table 3. In order to validate the simulation of the extracellular dehydration, predictions for two parameters were compared to measurements carried out on rats in the same range of weight (245 ± 2 g, n = 18). The experimental data indicate that ECF [Na^+^] measured at the end of the experimental protocol (136 ± 3 mmol/L), as well as water intake measured 4 and 24 h after the start of the protocol (2.8 ± 2 ml/100 g bw and 17.5 ± 3.5 ml/100 g bw, respectively) are all within the range of the predicted values.

**Table 3:** Predictions of the hydromineral parameters obtained with the simulated extracellular dehydration protocol (furosemide injections combined with a Na^+ ^deficient diet).

Parameters	First 4 h (day 0) Control	First 4 h (day 1) Extracellular dehydration	24 h (day 0) Control	24 h (day 1) Extracellular dehydration
Mean ECF [Na^+^] over the period (mmol/L)	141.6	146.8	141.8	141.3

Mean [AVP] (pg/ml)	2.2	4.5	2.7	4.8

Mean ECF Volume (ml/100 g)	22.7	22.0	22.7	21.7

Mean ICF Volume (ml/100 g)	45.5	44.0	45.4	45.6

ECF [Na^+^] at the end of the period (mmol/L)	141.8	149.9	142.2	134.0

Cumulative Urine Na^+ ^(mmol/100 g)	0.1	0.3	0.83	1.19

Water intake (ml/100 g)	0.1	3.5*	18.2	20.3

Na^+ ^intake (mmol/100 g)	0.0072	0	0.74	0.41

Urine Volume (ml/100 g)	1.8	4.1	12.4	12.8

Predictions at different time-points (4 h and 24 h) under control condition are listed under day 0 and predictions under Na^+ ^deficit are listed under day 1. "*" indicates the amount of water ingested during the first 4 h after access to tap water (see the method for a detailed description of the protocol).

## Discussion

The high temporal resolution of the graphical output of the simulation gives new insights into the hydromineral dynamic of the rat and allows the simulation to answer fundamental questions about the homeostatic parameters. The model integrates experimental data of different forms into specific meta-boxes to translate the biological realities into equations (i.e., [Na^+^]_urine_) or computational algorithms (i.e., the motivation module). Here, we discuss some of the strategic choices we made while building our model.

### Assumptions on the inputs to the body fluid compartments

Our model requires a "digestive module" sub-system to simulate the obligatory delays between fluid and Na^+ ^intake and their absorption by the body. For that purpose we used the time constants reported in a previous computer simulation of the water balance [12]. In this model, the water and Na^+ ^flow rates between digestive compartments were calculated from the water and [Na^+^] content of the stomach and intestine [27,28]. The model also includes passive and active transfer of water and electrolytes across the intestine wall. Various attempts were made to increase the accuracy of this module. For example, a second "digestive module" based on the recent study of Smith *et al*. (2007) was also tested in the model. However, the assumption of a constant rate (mmol/s) for sodium transfer between the digestive compartments [37] does not generate overall predictions in agreement with experimental data.

The model includes need-induced fluid intake, which is triggered when ECF [Na^+^] exceeds the pre-established set-point by 4%. Introducing this threshold in the model is based on two distinct studies [5,29]. In Anderson *et al*. (1990), the authors showed that a mean increase of 10 mosmol/L was needed to trigger drinking in pigs. This increase represents about 4% of the normal osmolality (289 mosmol/L). In the second study performed in humans, the authors presented a relationship between plasma osmolality and thirst sensation [5]. According to their regression function, a 4% increase in osmolality (298 mosmol/L vs. 287 mosmol/L) lead to a score of 5 over 10 in term of thirst sensation.

The "motivation" module of our model includes unregulated drinking, an essential aspect of fluid and electrolyte intake. Water and Na^+ ^consumption exceed the daily needs of the rat and physiological stimuli do not stop unregulated drinking [30]. This aspect of fluid intake was therefore introduced in our model to enhance the realism of the prediction generated by the simulation. Moreover, a circadian modulation of unregulated drinking was added to the model. This modulation takes into account the fact that the probability of drinking is not constant over 24 h and the probability of drinking was therefore modeled by a sinusoidal function of time with a minimum at 2 PM and a maximum at 2 AM. The maximum probability was set to the middle of the waking period (2 AM) in agreement with the fact that drinking is correlated with eating [38] and that rats almost exclusively eat during their waking period (night; [36]).

### Assumptions on the outputs to the body fluid compartments

It is known that the rate of renal Na^+ ^clearance can be slightly affected by hormones such as AVP [39,40]. However, the absence of suitable data on Na^+ ^clearance as a function of AVP concentration and the fact that the effect is negligible in most condition [9] led us to consider a non-variable parameter in the model. This non-variable Na^+ ^clearance does not imply a fixed Na^+ ^excretion in the model. Indeed, variable urine Na^+ ^excretion mechanism was introduced to control ECF [Na^+^] and volume (ANPs factor), as well as a Na^+ ^retention mechanism (ALD factor). The actions of these mechanisms combined with the action of the AVP controller exert a powerful mechanism of urine concentration.

AVP is the main controller of the model. The measurable AVP concentration in the plasma represents the equilibrium between AVP secreted from the pituitary gland and its clearance [41]. However, in our model, those two components of the equilibrium are not simulated individually, only the resulting AVP level. In order to have a more realistic simulation, we included the delay imposed by the fixed clearance of AVP. When AVP secretion is reduced, an exponential decay of plasma AVP concentration was introduced in our model. This decay was adjusted so that 15% of circulating AVP is cleared every minute [18] until the correct AVP level is reached. In addition, a recent study evaluating the circadian changes of plasma vasopressin in patients presenting nocturia, has reported a circadian modulation of AVP [42]. Inspired by this paper, a modulation of AVP was introduced in the model by assuming that a mechanism lowers the AVP level before the sleeping period of the rat in order to prevent nocturia. Based on Figure one of Graugaard-Jensen *et al*. 2006 (but with a 12 hours phase since rat are active during night and rest during the day), we introduced the following modulation: at 2 AM, the level of AVP is reduced in a linear function of time until a nadir of -30% is reached at 8 AM. Then, AVP concentration is restored to its computed level by 11 AM. It should be noted that the lack of these two regulatory mechanisms controlling the level of circulating AVP greatly impairs the quantitative and dynamic predictions of this parameter and resulting ECF [Na^+^].

### Focus on the long-term simulation of hydromineral balance

In the present study, the predictions mainly concern six individual parameters characterizing the hydromineral parameters and homeostatic responses: The ECF [Na^+^] and volume, the ICF volume, the AVP secretion and the cumulative water intake and urine excretion. We simulated normal hydromineral conditions to validate our model in terms of a quantitative prediction of the parameters of interest. Comparison of these predictions with published experimental data (Table 2) allows us to conclude that our model contains at least the minimal and essential sub-systems that are required to model the hydromineral balance. Moreover, these subsystems are correctly connected using appropriate approximations and time constants to produce overall realistic predictions. Therefore, the present high-level model is appropriate to deliver realistic simulation of AVP secretion and ECF osmolality under normal hydromineral conditions, i.e. when the rat meets regular conditions of laboratory housing. This latter assumption might reduce the precision of any predictions on the biological parameters of interest made under either pathophysiological conditions, or extreme experimental conditions (hemorrhage, severe dehydration) as these specific situations require the implementation of additional regulatory systems.

The simulation over 20 consecutive days predicts relatively large inter-day fluctuations in AVP, in water and electrolyte intake and in urine excretion compared to the stability of the ECF and the ICF parameters (see Table 2). These fluctuations indicate that the daily distribution of fluid and electrolyte intake under normal osmotic conditions is not completely correlated with physiological needs. On the contrary, the fluctuations might reflect the unregulated nature of water intake and suggests that either a deficit, or overload in fluid and Na^+ ^consumption occur on a daily basis. These large discrepancies may explain the inter-individual variability that has been reported in experimental data. For example, measurement of the urine flow rate and urine osmolality in a population of male Sprague-Dawley rats extended over an almost threefold range (Figure three in Bankir, 2001).

The long-term simulation produced mean data and smoothed the random variations in each parameter tested, leading to the identification of two distinct phases during the active period of the rat: the restoration and the maintenance. The identification of these two phases was based on the prediction of a time-dependent secretion of AVP that is well correlated with the phases of the hydromineral cycle. The predicted dynamics of plasma AVP shows a constant rise over the sleeping period of the rat (daylight) and a decrease during the active period of the rat (night). This pattern of secretion matches the partial experimental observations indicating a circadian pattern in plasma vasopressin level with a peak in secretion at the end of the sleeping period [11,35,36]. However, the temporal resolution of the experimental measurements is not as accurate as the simulation (4 measurements for Windle et al. 1992 and 6 for Granda et al., 1998 compared to 1440 for the simulation) and the estimation of the mean experimental AVP level and peak concentration for 24 h might be biased by individual fluctuations as shown in the Additional file 3. The temporal resolution of the simulation, as well as the average of AVP level at each time point for several consecutive days greatly improves our perception of the circadian secretion of AVP.

Interestingly, the simulation allows the prediction (quantification and dynamic) of parameters that would be difficult to measure experimentally, such as the ICF and ECF volume. Here, the long-term simulation highlights the role of the ICF compartment in the stabilization of the ECF volume. The sleeping period is associated with a progressive decrease in the ECF volume that is concomitant to the decrease in the ICF volume. The parallel depletion of both fluid compartments suggests the importance of water transfer between the ICF and the ECF compartment to attenuate the depletion of the extracellular compartment. The role of the ICF compartment was further demonstrated using a 24-h simulation, during which fluid exchange was blocked. Under these conditions, the threshold for drinking was reached more often and small random perturbations in the ECF [Na^+^] or AVP caused by drinking became larger. It should be noted that the role of the ICF compartment as a buffer is a primary and fast compensatory response triggered by all the simulated hydromineral challenges. The predicted reduction in the ECF volume during the night (maximum 7%) is certainly caused by evaporative water loss, which is compensated neither by water intake, nor by metabolic water. However, such a change in volume is not necessarily associated with hypotension. Indeed, blood pressure is regulated by additional mechanisms like vasoconstriction.

### Focus on the simulations of hydromineral challenges

The simulations of the hydromineral challenges yielded several important results. The simulation of intracellular dehydration indicates that restoration of the ECF [Na^+^] mainly involves two sets of responses with different dynamics. Water transfer from the ICF to the ECF, and AVP secretion are rapid homeostatic responses. Need-induced water intake is delayed and has a high impact on both the ICF volume and ECF [Na^+^]. However, avid drinking creates hypervolemic conditions and further restoration of the ECF volume involves water and salt excretion that extends over the next 6 h.

The simulation of furosemide injection led to predictions that are in agreement with the experimental data. It also indicates that hyponatremia is only achieved by combining diuretic injections with a Na^+ ^deficient diet (134.0 mmol/L: predicted ECF [Na^+^] vs. 136 ± 3 mmol/L: measured ECF [Na^+^] from our experimental protocol). Hyponatremia is correlated with a sustained increase in the ICF volume caused by the osmotic gradient generated between the ECF [Na^+^] and ICF [K^+^]. Furosemide injections *per se *induced hypernatremia, likely resulting from hyponatriuria. Experimental data showed that blocking Na^+ ^re-uptake with furosemide injections led to urine [Na^+^] ranging from slightly hyponatriuric to hyponatriuric (approx. 120 ± 20 mmol in [43]; approx. 107 ± 22 in [44]; approx. 80 mmol in [31]). Despite a larger hyponatriuria, the data reported in Hori et al., 1988 (Figure oneA) were privileged because they provided the most detailed experimental measurements needed to create an accurate furosemide model. It should be noted that implementation of a higher Na^+ ^excretion rate in the furosemide model would lower ECF Na^+ ^content without creating hyponatremia. In this line, we tested the assumption in which we forced isonatriuria, approximately 140 mmol/L Na^+^, during the simulated furosemide treatment. This extreme simulation also led to slightly hypernatremic ECF after the first 4 h of simulation (data not shown), suggesting that furosemide treatment *per se *is not sufficient to cause efficient Na^+ ^depletion of the ECF compartment.

## Conclusion

In the present study, we developed a high-level simulation of hydromineral homeostasis. The coherence shown between the simulations (quantification, dynamics) and the experimental data indicates that the model integrates most of the essential components to predict realistic physiological values for the parameters under study. Therefore, the model is able to render quantitative and precise temporal predictions on the ECF osmolality and AVP secretion, as well as on their relationship with the ECF and ICF volume, making it an appropriate tool to predict the state of the rat hydromineral balance when challenged by various osmotic perturbations. Such predictions will help in the design of experimental protocols to test specific hypothesis. For example, we can suggest that the best period to experiment on rat is during the maintenance phase as this is the period where hydromineral fluctuations are less likely. Also, obtaining a global view of all the hydromineral parameters that the simulation can produce with equivalent temporal precision would be difficult or impossible to obtain with in vivo measurements. Moreover, one interesting application of the model is its use in predicting the putative action of pharmacological compounds on the hydromineral balance. As an example, we use the present model to simulate furosemide injections and our results are compatible with experimental data demonstrating that our model would be appropriate to test the action of other pharmacological compound affecting the homeostasis in the future.

The use of the model to an extended range of applications requires however, the implementation of additional components. For example, the implementation of a graded Na^+ ^appetite would be possible by modeling a sophisticated renin-angiotensin-aldosterone system and digestive module in the model. Interestingly, substitution of the meta-box "vasopressin level" by a biologically inspired simulation of the magnocellular neuron network [45] in future versions of the simulation would allow us to predict how local modulation of neuronal excitability by osmotic challenges and drugs targeting these neurons would influence the dynamics of global homeostatic parameters in rats.

## Authors' contributions

LN established the rat model and programmed the simulation using computer software. He wrote the first draft of the present paper and he approves the final version. DA performed the animal experiments, i.e. the furosemide injections and all the measurements of the parameters. Moreover she participated to the criticisms and revisions of the manuscript. DA approved the last version of the manuscript. DM analyzed the experimental and predicted data of the simulation and he suggested all the final improvements of the model. He performed all the writing revisions of the manuscript and approved the last version of the manuscript.

## Supplementary Material

Additional file 1 **Comparison of the impact of osmotic and volemic fluctuation on vasopressin secretion**. This additional file shows the relative impact of the osmotic and volemic fluctuation on the AVP secretion based on equation 1 of the method. Click here for file

Additional file 2 **Calculation of the sodium clearance used in the simulation**. Four different calculations of the sodium clearance based on four different paper are presented and their results are averaged in order to generate the sodium clearance value used in the simulation. Click here for file

Additional file 3 **Comparison of the simulated dynamics of three hydromineral parameters with experimental data**. This additional file compared the simulated dynamics of the water intake (input), AVP concentration (controller) and urine volume (output) with experimental data and shows that they are in agreement. Click here for file
