# Supplementary material for: Computational simulation of vasopressin secretion using a rat model of the water and electrolyte homeostasis
Source: BMC Physiol. 2010 Aug 25;10:17. doi: 10.1186/1472-6793-10-17 (PMC2939538; doi:10.1186/1472-6793-10-17)
Supplement: Additional file 1 — Comparison of the impact of osmotic and volemic fluctuation on vasopressin secretion. This additional file shows the relative impact of the osmotic and volemic fluctuation on the AVP secretion based on equation 1 of the method. [file 1472-6793-10-17-S1.PDF]

Appendix 1: Comparison of the impact of osmotic and volemic fluctuation on vasopressin secretion.

**AVP secretion (pg/ml)**						
**Conditions**		**Osmotic Contribution**			**Volemic Contribution**
**[Na^+] (%)**	**ECF_V (%)**	**Δax**	**Δbx**	**Absolute total |Δ(a+b)x|**	**Δc**
**Hyponatremia -2.86%**	**-2.86**	-0.728	-0.094	0.822	0.814
	**0**	-0.728	0.000	0.728	0.000
	**2.86**	-0.728	0.094	0.634	0.000
**Normal**	**-2.86**	0.000	0.157	0.157	0.814
	**0**	0.000	0.000	0.000	0.000
	**2.86**	0.000	-0.157	0.157	0.000
**Hypernatremia +2.86%**	**-2.86**	0.728	0.409	1.137	0.814
	**0**	0.728	0.000	0.728	0.000
	**2.86**	0.728	0.409	0.319	0.000

**AVP (pg/ml)**					
**Conditions**		**Osmotic Contribution**			**Volemic Contribution**
**[Na^+] (%)**	**ECF_V (%)**	**Δax**	**Δbx**	**Absolute total |Δ(a+b)x|**	**Δc**
**Hyponatremia -4%**	**-4**	-1.019	-0.273	1.292	1.266
	**0**	-1.019	0.000	1.019	0.000
	**4**	-1.019	0.273	0.746	0.000
**Normal**	**-4**	0.000	0.220	0.220	1.266
	**0**	0.000	0.000	0.455	0.000
	**4**	0.000	-0.220	0.220	0.000
**Hypernatremia +4%**	**-4**	1.019	0.713	1.732	1.266
	**0**	1.019	0.000	1.019	0.000
	**4**	1.019	-0.713	0.306	0.000

The appendix shows the relative impact of variations in extracellular Na^+ level (osmolality) and volume on the secretion of AVP, based on equation 1 presented in the methods. This equation can be expressed as $y = (a+b)x + c$ with:
$a = 0.91$

$$b = -11\frac{ECF_{\Delta V}(t)}{ECF_V(t=0)}$$

$x = 200([Na^{+}{}_{ECF}] - 0.1375)$

$$c = 1.3 \cdot e^{-17\frac{ECF_{\Delta V}(t)}{ECF_V(t=0)}}$$

The first condition (upper table) compares a change of ±2.86% of ECF [Na^+] (±4 mmol Na^+) and a change of ±2.86% of ECF volume on AVP secretion. This condition represents typical daily fluctuation of ECF [Na^+] (142 mmol: daily mean vs. 145 mmol: mean [Na^+] peak). The second condition (lower table) compares a change of ±4% of ECF [Na^+] (±5.6 mmol Na^+) and a change of ±4% of ECF volume on AVP secretion. This condition represents the change in ECF [Na^+] that was set in the model as the threshold for drinking. The baseline value for each term in equation 1 (obtained with 0% change in ECF [Na^+] and volume) was subtracted to enhance the relative impact of the changes on AVP secretion.
