# Supplementary material for: Computational simulation of vasopressin secretion using a rat model of the water and electrolyte homeostasis
Source: BMC Physiol. 2010 Aug 25;10:17. doi: 10.1186/1472-6793-10-17 (PMC2939538; doi:10.1186/1472-6793-10-17)
Supplement: Additional file 2 — Calculation of the sodium clearance used in the simulation. Four different calculations of the sodium clearance based on four different paper are presented and their results are averaged in order to generate the sodium clearance value used in the simulation. [file 1472-6793-10-17-S2.PDF]

Appendix 2: Calculation of the fixed sodium clearance for the simulation

Symbol	Description
C_{Na^+}	Sodium clearance
C_{Cr}	Creatinine clearance
FE_{Na^+}	Sodium fractional excretion
GFR	Glomerular filtration rate
$[Na^+_{ECF}]$	ECF sodium concentration
Na^+_{Urine}	Urine sodium excretion rate

Ahloulay et al. 1999
From Table 1 :

$$Na^+_{Urine} = 2.54 \pm 0.34 (mmol/day \cdot 330g\ bw) = 0.00054 \pm 0.00007 (mmol/min \cdot 100g\ bw) \tag{1}$$

In this study the author indicates that plasma $[Na^+]$ was normal (no value given). Therefore, we used a wide $[Na^+_{ECF}]$ in the normal range :

$$[Na^+_{ECF}] = 0.140 \pm 0.005 (mmol/ml) \tag{2}$$

The maximal and minimal Na^+ clearance is computed as followed :

$$Max(C_{Na^+}) = Max(Na^+_{Urine})/Min([Na^+_{ECF}]) = 0.0045 (ml/min \cdot 100g\ bw) \tag{3}$$

$$Min(C_{Na^+}) = Min(Na^+_{Urine})/Max([Na^+_{ECF}]) = 0.0032 (ml/min \cdot 100g\ bw) \tag{4}$$

For a rat of 255 g :

$$Max(C_{Na^+}) = 0.012 (ml/min) \text{ and } Min(C_{Na^+}) = 0.0083 (ml/min) \tag{5}$$

Calculated C_{Na^+} of :

$$\boxed{C_{Na^+} = 10.15 \pm 1.85 (\mu l/min)} \tag{6}$$

Boer et al. 2005
Estimation from Figure 2 :

$$FE_{Na^+} = 0.008 \pm 0.001 \tag{7}$$

$$C_{Cr} = 360 \pm 15 (\mu l/min \cdot 100g\ bw) \tag{8}$$

The maximal and minimal Na^+ clearance is computed as followed :

$$Max(C_{Na^+}) = Max(FE_{Na^+}) \cdot Max(GFR) = 0.009 \cdot 375 = 3.3 (\mu l/min \cdot 100g\ bw) \tag{9}$$

$$Min(C_{Na^+}) = Min(FE_{Na^+}) \cdot Min(GFR) = 0.007 \cdot 345 = 2.4 (\mu l/min \cdot 100g\ bw) \tag{10}$$

For a rat of 255 g :

$$Max(C_{Na^+}) = 0.0086 (ml/min) \text{ and } Min(C_{Na^+}) = 0.0062 (ml/min) \tag{11}$$

Calculated C_{Na^+} of :

$$\boxed{C_{Na^+} = 7.4 \pm 1.2 (\mu l/min)} \tag{12}$$

Jonassen et al. 2000
From Table 3 :

$$FE_{Na^+} = 0.0034 \pm 0.0003 \tag{13}$$

From Table 2 :

$$GFR = 962 \pm 58 (\mu l/min \cdot 100g\ bw) \tag{14}$$

The maximal and minimal Na^+ clearance is computed as followed :

$$Max(C_{Na^+}) = Max(FE_{Na^+}) \cdot Max(GFR) = 0.0037 \cdot 1020 = 3.8 (\mu l/min \cdot 100g\ bw) \tag{15}$$

$$Min(C_{Na^+}) = Min(FE_{Na^+}) \cdot Min(GFR) = 0.0031 \cdot 904 = 2.8 (\mu l/min \cdot 100g\ bw) \tag{16}$$

For a rat of 255 g :

$$Max(C_{Na^+}) = 0.0096 (ml/min) \text{ and } Min(C_{Na^+}) = 0.0071 (ml/min) \tag{17}$$

Calculated C_{Na^+} of :

$$\boxed{C_{Na^+} = 8.35 \pm 1.25 (\mu l/min)} \tag{18}$$

Shirley et al. 1994
From Figure 1 :

$$Na^+_{Urine} = 1.8 \pm 0.1 (mmol/day) = 0.00125 \pm 0.00007 (mmol/min) \tag{19}$$

From Table 1 :

$$[Na^+_{ECF}] = 0.147 \pm 0.001 (mmol/ml) \tag{20}$$

The maximal and minimal Na^+ clearance allowed is computed as followed :

$$Max(C_{Na^+}) = Max(Na^+_{Urine})/Min([Na^+_{ECF}]) = 0.0090(ml/min) \tag{21}$$

$$Min(C_{Na^+}) = Min(Na^+_{Urine})/Max([Na^+_{ECF}]) = 0.0080(ml/min) \tag{22}$$

The value was not scaled to weight because the rat weight used in the model is in the range of the animal weight used in this study (245 ± 4 g to 264 ± 5)
Calculated C_{Na^+} of :

$$\boxed{C_{Na^+} = 8.5 \pm 0.5(\mu l/min)} \tag{23}$$

Sodium clearance value used in the simulation
The mean of the calculated values of C_{Na^+} is used in the model : $(10.15 + 8.35 + 7.4 + 8.5)/4 = \boxed{8.6\mu l/min}$. This fixed value of C_{Na^+} is well within the permissible range calculated in the four studies of reference.
