# Supplementary material for: Computational simulation of vasopressin secretion using a rat model of the water and electrolyte homeostasis
Source: BMC Physiol. 2010 Aug 25;10:17. doi: 10.1186/1472-6793-10-17 (PMC2939538; doi:10.1186/1472-6793-10-17)
Supplement: Additional file 3 — Comparison of the simulated dynamics of three hydromineral parameters with experimental data. This additional file compared the simulated dynamics of the water intake (input), AVP concentration (controller) and urine volume (output) with experimental data and shows that they are in agreement. [file 1472-6793-10-17-S3.PDF]

Appendix 3: Comparison of the simulated dynamics of three hydromineral parameters with experimental data

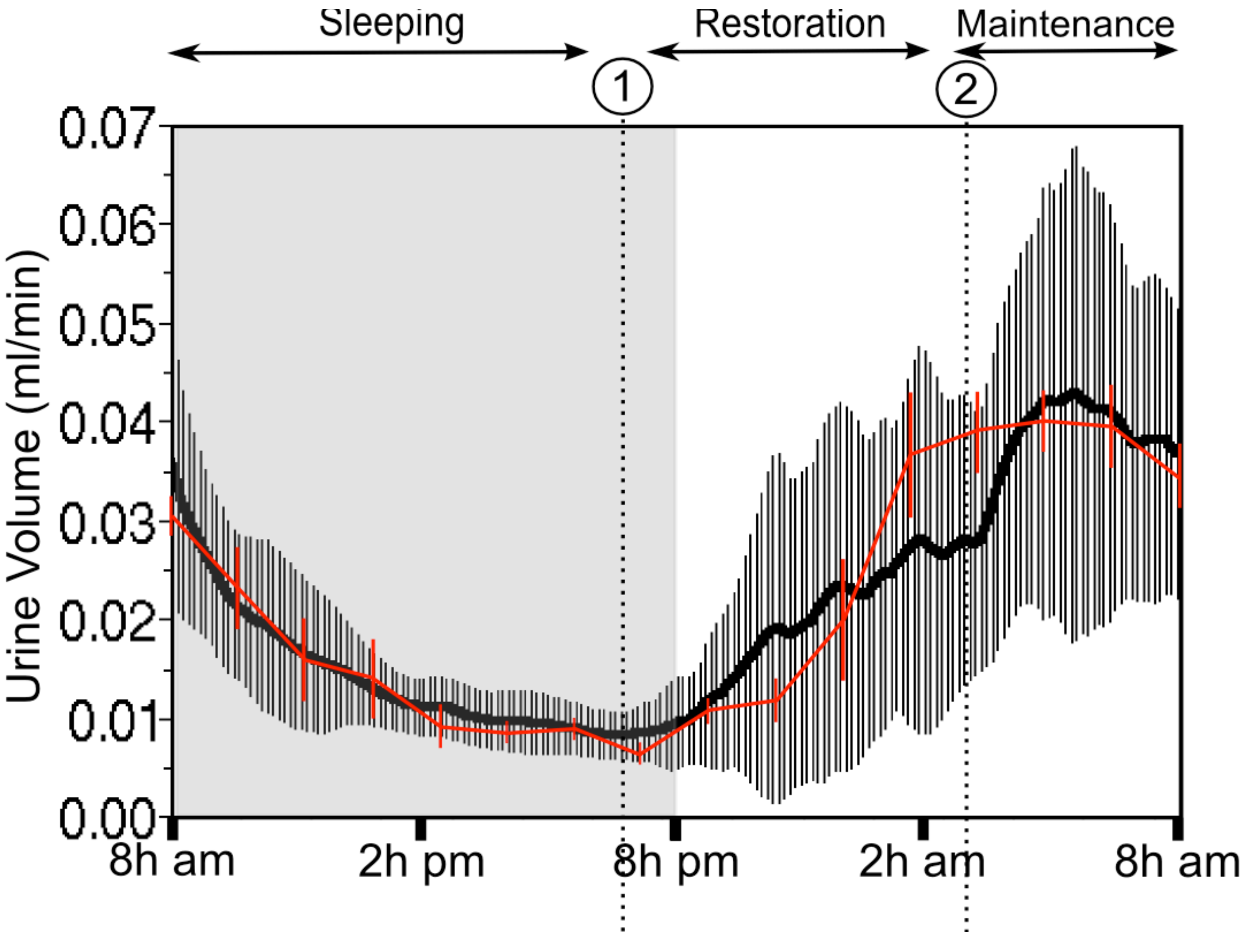

A) Validation of the predicted dynamic of the urine volume.

The thick black line and bars simulate the mean ± SD of the urine volume over 20 consecutive days. The thin red line and bars illustrate the experimental measurements of the urine volume for 24h (mean ± SE) obtained from Aizman et al. 1994 (redrawn from Figure 2 with permission from APS: Identifier R393-3). The experimental measurements (16 time points) fit precisely within one order of SD of the predicted value.

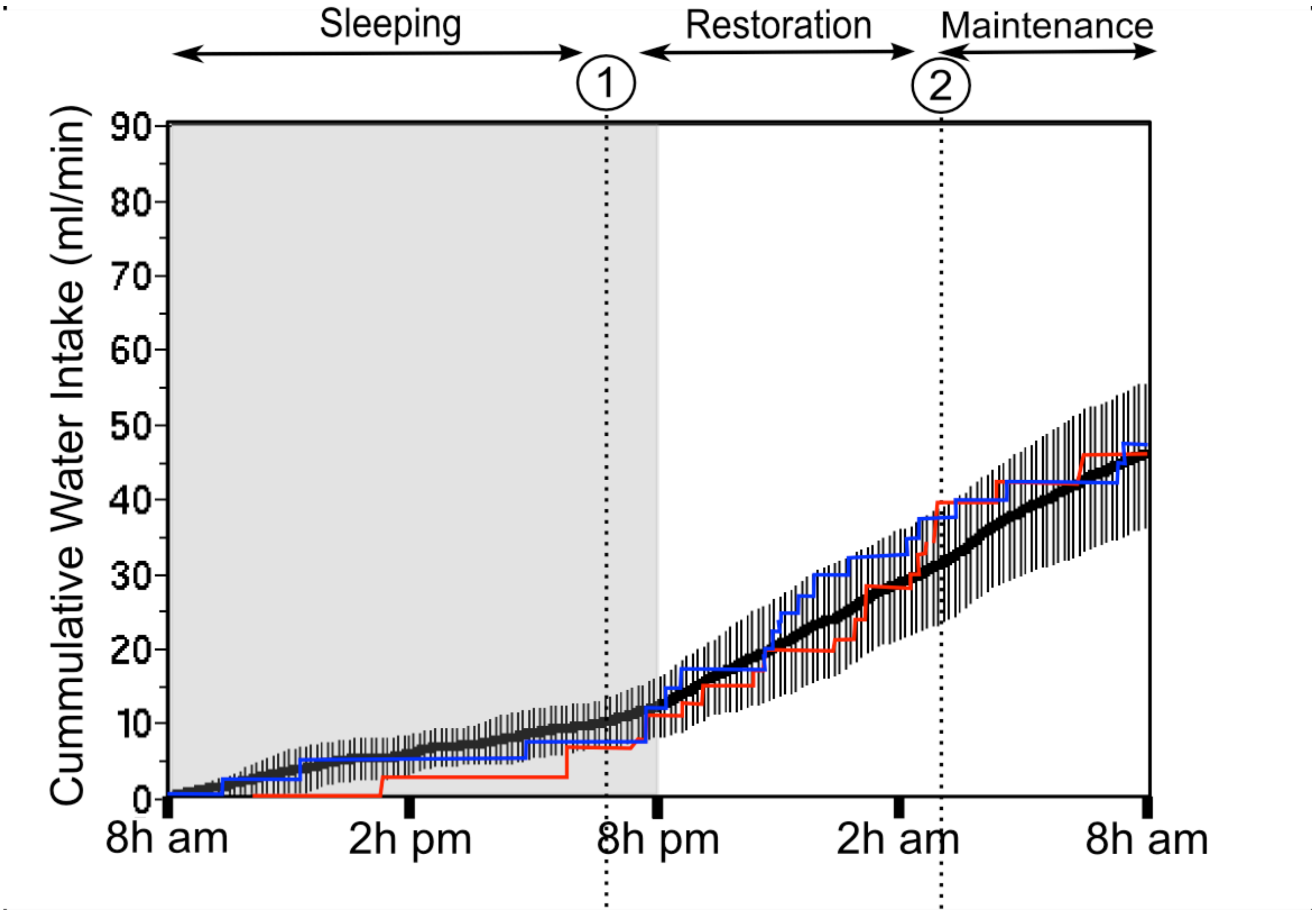

B) Validation of the predicted dynamic of cumulative water intake. The thick black line and bars simulate the the mean ± SD of cumulative water intake over 20 consecutive days. The blue line illustrates one particular trajectory of the simulation. The red line illustrates experimental data obtained from Fitzsimons et al. 1969 (7 rats). These data were linearly rescaled so that the last experimental and predicted value (8h AM, right) is identical. The small discrepancy between the experimental and simulated data observed during the sleeping period is likely caused by the unmatched number of drinking bouts that occur during the period (see results for details).

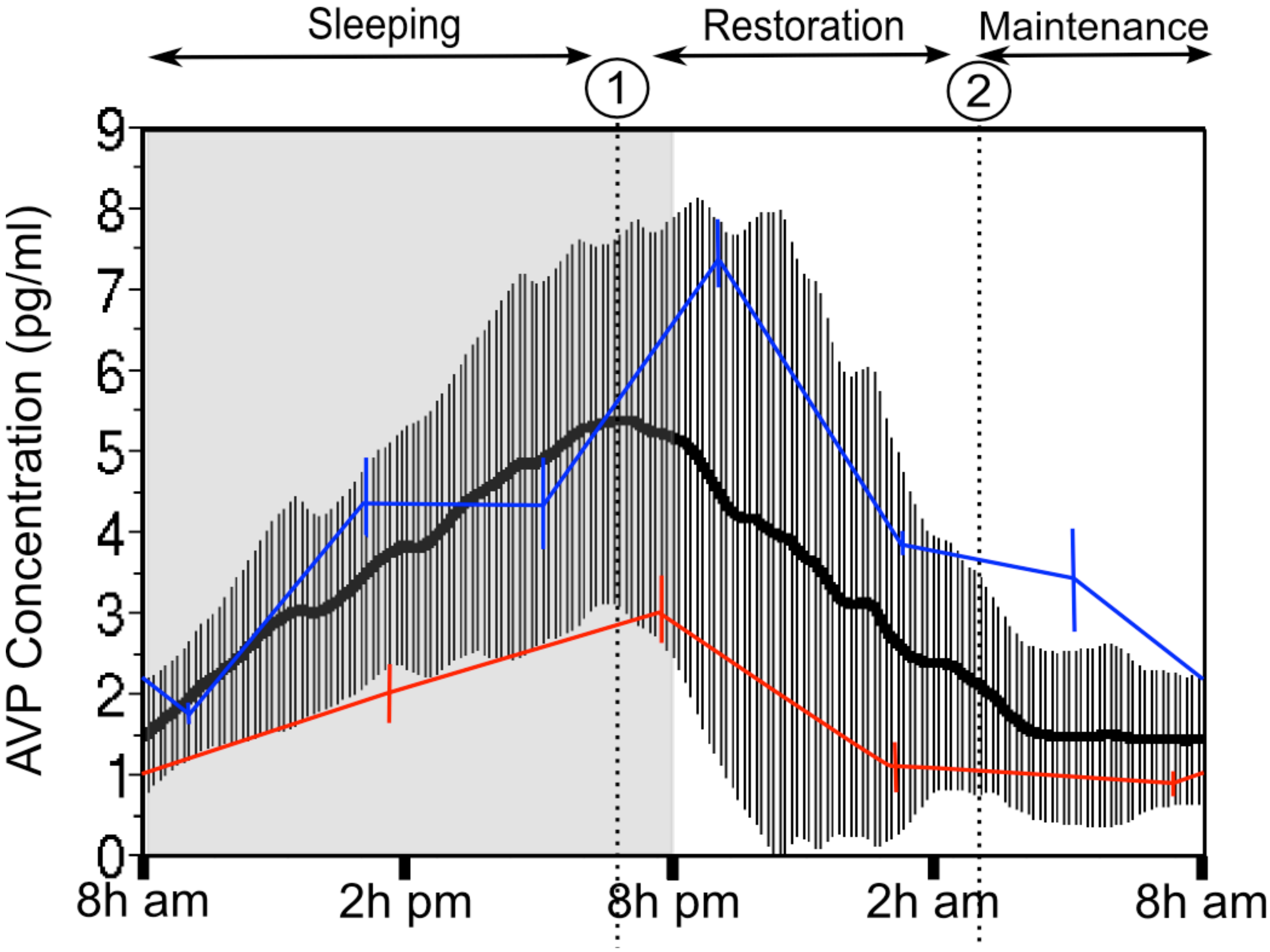

C) Validation of the predicted dynamic of AVP concentration. The thick black line and bars simulate the mean ± SD of AVP concentration over 20 consecutive days. The blue and red line illustrates experimental measurements (24h) obtained from Granda et al. 1998 (6 time points; with permission from Elsevier; license # 2473100864208) and from Windle et al. 1992, respectively (with permission from the Society for Endocrinology). These data illustrate the variability of the experimental data and validate the circadian fluctuation of the simulated AVP secretion.
